# Challenges of identification and anonymity in time-continuous data from medical environments

**DOI:** 10.3389/fdgth.2025.1604001

**Published:** 2025-08-20

**Authors:** Freimut Hammer, Thorsten Strufe

**Affiliations:** KASTEL Security Research Labs, Karlsruhe Institute of Technology (KIT), Karlsruhe, Germany

**Keywords:** privacy, medical data, differential privacy (DP), anonymity, data sharing and reuse

## Abstract

In medical environments, time-continuous data, such as electrocardiographic records, necessitates a distinct approach to anonymization due to the paramount importance of preserving its spatio-temporal integrity for optimal utility. A wide array of data types, characterized by their high sensitivity to the patient’s well-being and their substantial interest to researchers, are generated. A significant proportion of this data may be of interest to researchers beyond the original purposes for which it was collected. This necessity underscores the pressing need for effective anonymization methods, a challenge that existing approaches often fail to adequately address. Robust privacy mechanisms are essential to uphold patient rights and ensure informed consent, particularly within the framework of the European Health Data Space. This paper explores the challenges and opportunities inherent in developing a novel approach to anonymize such data and devise suitable metrics to assess the efficacy of anonymization. One promising approach is the adoption of differential privacy to account for temporal context and correlations, making it suitable for time-continuous data.

## 1 Introduction

Patient data is defined as physiological data and metadata collected in a medical environment. This paper concentrates on this subject, with implications that extend to time-continuous data in general. Such data can be recorded in a variety of settings, including hospitals, research facilities, and clinical practices. In a broader sense, physiological data collected by wearable devices such as smartwatches can also be considered patient data. This data encompasses information related to an individual’s physiology, psychology, and overall health status, facilitating a unique identification. Due to its sensitivity, such data necessitates a regulatory framework that ensures it is handled with particular care.

The release or sharing of such data is essential for the generation of knowledge with mutual control and replicability and is therefore fundamental to the ethics and progress of science.

The utilization of patient data can facilitate the detection of rare diseases or serve as realistic training material for medical professionals. Additionally, it facilitates the training and verification of machine-learned models for medical data and enables data-driven research in this field in general.

These objectives are often in stark contrast with the sensitivity of the data, especially in medical environments. This underscores the necessity for a mechanism to safeguard the individual behind the data. A mechanism that effectively anonymizes data would be an optimal solution, enabling straightforward sharing of data among medical professionals or researchers, as well as its publication, without compromising the rights of the individual to whom the data pertains.

**Figure 1:**
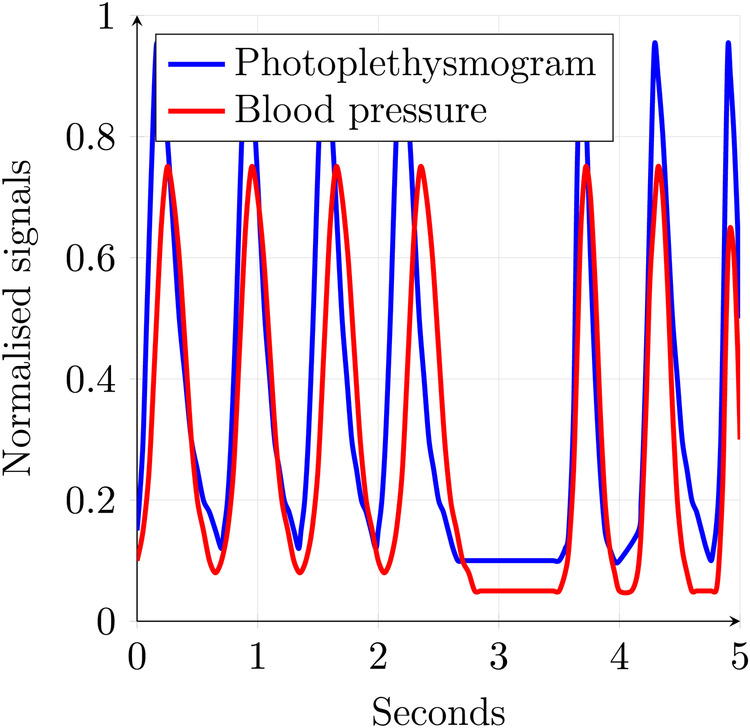
Time-continuous medical data example.

A distinguishing feature of numerous categories of medical data is their time-continuous nature. The spatio-temporal relationship within such data is of paramount importance to their utility. An example of such data is an ECG, which can be seen in Figures 4–6. There are plenty of different types of such data, like SPO${}_{2}$ saturation, electroencephalogram, and blood pressure. In Figure 1, two physiological signals—photoplethysmogram (PPG) and arterial blood pressure (BP)—are plotted over time to enable direct visual comparison of their temporal dynamics. The data is derived from Wikimedia Commons (1). To facilitate this comparison, both signals have been normalized to a common scale between 0 and 1 along the y-axis. This normalization preserves the relative shape and timing of signal fluctuations while removing the influence of differing absolute amplitudes and units. The blue line represents the PPG signal, which reflects blood volume changes in the microvascular bed of tissue, typically measured using optical sensors. It is characterized by sharp upstrokes corresponding to systolic blood ejection, followed by a slower decay during diastole. The red line represents the arterial blood pressure waveform, which reflects pressure changes within the arteries during the cardiac cycle. It features broader peaks and lower frequency content compared to the PPG signal. Plotting both signals on the same normalized axis reveals their phase relationship, morphological similarity, and temporal alignment, features that are especially useful in multimodal cardiovascular analysis, where understanding the interaction between pressure and volume dynamics is critical. Although normalization removes units (e.g., mmHg or arbitrary light absorption units), it retains the essential temporal characteristics needed for waveform analysis, including peak timing, rise and fall slopes, and periodicity.

Both ECG and the signals shown in Figures 1, 4–6 are time-continuous, as they reflect physiological processes—electrical activity, blood volume, and pressure—that evolve smoothly over time without discrete jumps. These signals are typically sampled at high frequencies to capture their continuous nature and preserve critical temporal features such as waveforms and phase relationships.

Formalizing and analyzing mechanisms to ensure privacy and utility is crucial for sensitive applications. Existing methods such as k-anonymity (2), t-closeness (3), and l-diversity (4) do not provide any information-theoretic guarantees or statements regarding the level of privacy achieved.

Implementations such as CASTLE (5, 6), which employs k-anonymity to continuous streams of non-continuous data, and SABRE (7), which utilizes a t-closeness-centered bucketization approach to achieve k-anonymity, are limited in their ability to preserve the spatio-temporal relation by compressing or stretching the data. Other approaches that preserve spatio-temporal relationships, like the group- and link-based approach by Nergiz et al. (8), fail to maintain the continuity, as the generation of representative data might not preserve the order or the step size. A notable shortcoming of the aforementioned approaches is the absence of formal statements about the level of privacy achieved.

Differential privacy (DP) stands out as a notable exception, offering a formal framework that provides robust guarantees and quantifies the level of privacy attained (9). The application of DP to health data has been examined by Dankar and El Emam (10). However, their analysis does not address the specific challenges posed by time-continuous data or the necessity of preserving spatio-temporal dependencies. Instead, their focus lies in the realm of categorical or numerical point-based measurements. In contrast, Olawoyin et al. (11) introduced a bottom-up generalization approach for temporal data. After the generalization of the time dimension, a DP mechanism is employed to ensure privacy along the value axis. This approach, while preserving the spatio-temporal relation, destroys the continuity of the data along the time axis.

This means that current approaches are not suitable for this task, as they fail to preserve the spatio-temporal relationships and the continuity of the data. Additionally, most fail to provide strong formal guarantees.

The ARX anonymization framework, developed at the Charité Berlin, is an open-source tool designed to protect sensitive data. It implements a wide range of de-identification algorithms and risk analysis methods (12). The ARX model has emerged as a promising candidate for seamlessly integrating a developed anonymization mechanism for time-continuous medical data. Formalizing a utility that is suitable for most, if not all, kinds of diagnoses and analyses that can be performed on the data can be challenging, as different diagnostic or analytical tasks depend on vastly different characteristics of the data. For instance, an ECG can be used by a medical professional to not only inspect various infractions by analyzing multiple parameters such as the lengths, amplitudes, and distances between recorded waveform complexes but also to assess ventricular depolarization by analyzing the electrical axis of the heart. This can be approximated by calculating the area under the curve of the QRS complex (13).

In this paper, we explore the intricate field of anonymizing time-continuous medical data. We emphasize the importance of addressing this issue and highlight the limitations of current methods. We elaborate the specific challenges a suitable mechanism must solve, the opportunities that arise from such a solution, and the requirements for potential quality metrics. To the best of our knowledge, no existing approach effectively anonymizes time-continuous medical data while preserving its utility for diagnostic and analytical purposes, such as investigating correlations between diagnoses and patient characteristics. This paper introduces and formalizes candidate metrics for utility and privacy. It also emphasizes the challenges a potential solution must address and the opportunities that arise from overcoming them.

## 2 Background

In this section, the foundation is established by formalizing the fundamental properties required for the anonymization of time-continuous medical data. This facilitates formal analysis of such properties and supports the evaluation of the usefulness of different classes of anonymization approaches, which are addressed in Section 3.

The notation is used to define the requirements, challenges, and opportunities for an anonymization approach targeting the class of data defined in Definition 2.6, which preserves the time continuity.

### 2.1 Notation

The date is considered part of a domain, where a point within this domain represents a data value or attribute, as formalized in Definition 2.1.

DEFINITION 2.1 **(Data value)**

A value v∈D, where D is the domain of this value, is called a data value. It is sometimes called an attribute.

An example of a data value is the height of the patient, e.g., 183 cm, or a continuous measurement of blood pressure.

Multiple data values corresponding to the same individual or case make up a data record, as formalized in Definition 2.2.

DEFINITION 2.2 **(Data record)**

A tuple $d\in D_{1}\times \cdots \times D_{n}$, where $D_{i}$ is the domain of the ith element and the tuple refers to exactly one individual, is called a data record. A data record is a cross-product of multiple data values. That is, $d=v_{1}\times \cdots \times v_{n}$, where $v_{i}$ is a data value.

The patientID, e.g., the hexadecimal ID de1dc2e6ead6, together with two data values from the previous example, i.e., height and continuous blood pressure, forms a simple data record.

Usually, multiple data records are processed together, forming a database, as formalized in Definition 2.3.

DEFINITION 2.3 **(Database)**

A set of data records $S\subseteq \{t_{1},\ldots t_{n}\}$ is called a database if each $t_{j}$ is a data record.

An example of such a database could be the following set of patients, where each patient is a data record:

• patientID: de1dc2e6ead6
  • height: 183 cm
  • continuous blood pressure: <waveform>
• patientID: b1f63e6de60d
  • height: 159 cm
  • weight: 93 kg
  • continuous blood pressure: <waveform>
  • email: johndoe@example.com
• patientID: cefddf2267dd
  • continuous blood pressure: <waveform>

Not all data records are required to contain the same types of data values, even though it is practical for them to do so in practice.

### 2.2 Continuity

Continuity can be seen as a property of the generating process. For example, the electrical activity of the heart, as measured by an ECG, is continuous according to Definition 2.5. However, it can also be a property of the recording process. When both the generating and recording processes are continuous, the resulting data value can also be considered truly continuous. As all data recorded by a system is somehow sampled and not truly continuous, another distinction is needed. This is provided in Definition 2.4.

DEFINITION 2.4 **(Variable and equidistant step time)**

Considering m data records of possibly different recordings $d_{i}=(t_{i},v_{i})$, where $t_{i}$ is the time and $v_{i}$ is the value, and $\Delta t_{n}=t_{n}-t_{n-1}$ for $n\in N_{>0}$.

If $\Delta t_{1}=\Delta t_{2}=\cdots =\Delta t_{m}$, then the time component of this data record has equidistant time steps; otherwise, it is considered a time attribute with variable step size. For multiple devices or recordings, the time attribute is said to have an equidistant step size if the Δt is equal for all devices or recordings.

**Figure 2:**
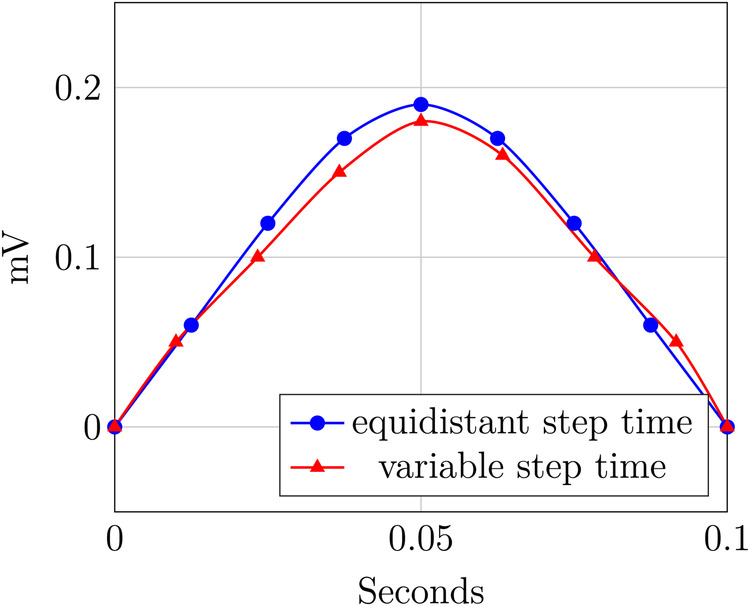
Variable and equidistant step time.

Figure 2 illustrates Definition 2.4. It shows two P-waves of an ECG: the blue curve has the same time intervals between measurements and thus exhibits equidistant time steps, whereas the red curve shows slight variations in the sampling rate and thus exhibits a variable time step.

We define continuity for data records and their databases with with a single time axis based on the ϵ,δ criterion for continuous functions by Weierstraß and Jordan, as follows:

DEFINITION 2.5 **(Continuity)**

Given a data record $d=(e_{0},e_{1},\ldots ,e_{n})\;|\;e_{i}=(t_{i},v_{i})\in T\times D$, where T is the time domain and D is some value domain. D must have subtraction and absolute operation defined on its elements. Additionally, the order operator has to define a total order on all non-negative elements of the domain.^1^ For every time entry of d, it holds that $t_{i}<t_{i+1}$.

We write that d contains t, or $t\in_{T}d$, if $t=t_{i}$ for some i∈[0,n].

Let $f\;:\;T\mapsto D;t\mapsto \left\{ \begin{array}{c} v_{i}\;:\;\text{if}\;t\in_{T}d\;\\ i(t)\;:\;\text{else}\; \end{array}\right.$, where i:T↦D is some sensible, domain- and application-specific interpolate. The data record d is continuous if f fulfills the ϵ,δ criterion. A database is called continuous if every record it contains is continuous.

**Figure 3:**
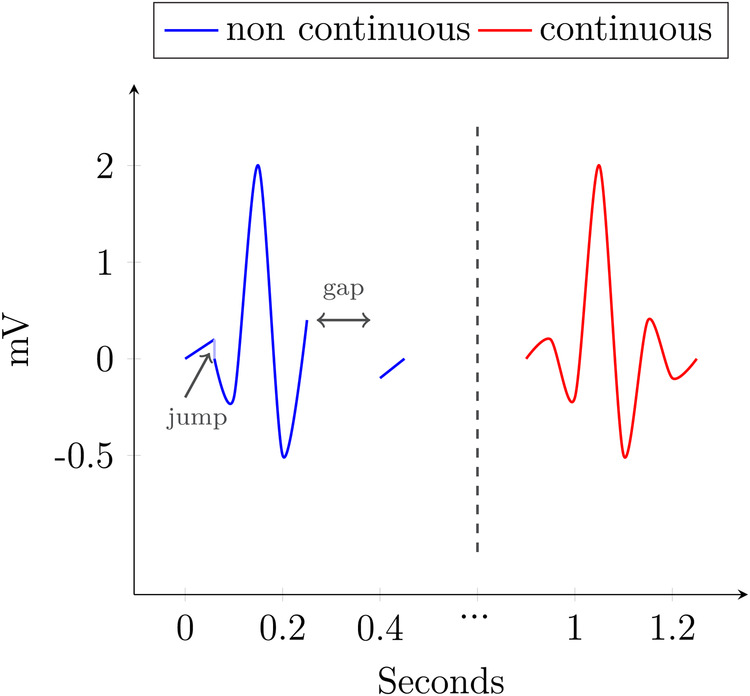
Continuity.

Figure 3 shows an example with two QRS complexes. The blue one contains a spot, where the value jumps from 1 mV to another at the same timepoint and includes a gap in the data, where no sensible interpolation is possible. The other, in red, is continuous.

For most practical applications, especially the class of data considered in this work, introduced formally in Definition 2.6, Definition 2.5 leads to Corollary 2.1.

COROLLARY 2.1 **(Practical continuity)**

To be considered continuous according to Definition 2.5, a data record—and by extension, a database—has to fulfill the following conditions:

1. The sampling rate needs to be high enough; i.e., the step size needs to be sufficiently small.
2. A heuristic to interpolate values between every possible pair of samples can be defined.
3. This interpolate introduces no discontinuities between the samples.

Conditions 2 and 3 depend on the recorded process, whereas Condition 1 depends on the recording process. This means that someone checking the requirement can look at the recording process and decide whether Condition 1 is fulfilled. However, for Conditions 2 and 3, it is necessary to find a suitable interpolate or provide proof of its absence. If Corollary 2.1 cannot be fulfilled, the data is not considered continuous.

Two exemplary interpolates are introduced in Equations 1 and 2. The interpolate $i_{1}$ in Equation 1 holds the last valid value until a new one is reached, whereas the interpolate $i_{2}$ in Equation 2 applies linear interpolation between two samples. $i_{1}$ is not a suitable candidate for any dataset that could potentially fulfill Corollary 2.1, as it produces jumps into the record, thus violating Condition 3 and the ϵ,δ criterion of Weierstraß and Jordan. In contrast, $i_{2}$ does not produce such jumps and is thus a valid candidate.

Equation 1 is well-defined for all time entries after the first sample and is undefined for all prior time points. In contrast, Equation 2 is well-defined for all time entries between the first and last sample, provided that at least two samples exist; otherwise, it is undefined.

(1) $$\begin{array}{rl} & i_{1}\;:\;T\mapsto D;t\mapsto \left\{ \begin{array}{c} v_{i}\;:\;\text{if}\;t\in_{T}d\;\\ i_{1}(t-1)\text{ else }\; \end{array}\right. \end{array}$$

(2) $$\begin{array}{rl} & i_{2}\;:\;T\mapsto D;t\mapsto \left\{ \begin{array}{c} v_{i}\;:\;\text{if}\;t\in_{T}d\;\\ \frac{i_{2}(t-1)+i_{2}(t+1)}{2}\text{ else }\; \end{array}\right. \end{array}$$

### 2.3 Class of data

There is a huge variety in medical time-continuous data types, many of which share some similar properties. As the general properties and requirements are identical, we focus on a small subset of such data without the loss of generality. Thus, the data used for the proposed approach in this work is that induced by a patient monitor and formalized in Definition 2.6. Patient monitor data is chosen due to its prevalence in both clinical setting and research databases like MIMIC IV (14) and eICU (15).

DEFINITION 2.6 **(Considered class of data)**

The class of data considered in this work is a database consisting of multiple domains, each linked by an identifier. Every domain consists of a time dimension and a value dimension, i.e., the domain $D=T\times D_{v}$. Both dimensions have to fulfill continuity criteria according to Definition 2.5. The value dimension typically consists of a series of real values, which are generated by events resampling a waveform, e.g., an ECG, and sometimes represents a numeric value, e.g., SPO${}_{2}$.

Many databases of this type have already been published. For example, MIMIC IV v.3.1 contains data from 364,627 individuals and 546,028 hospitalizations from emergency and intensive care units (14). Another example is the PTB-XL database, which contains 21,799 clinical 12-lead ECGs from 18,869 individuals, each lasting 10 s. Every ECG in the PTB-XL database is annotated by one or two cardiologists (16, 17). One example of a database with a broader scope is eICU. It collects data from Philips eICU monitors in multiple intensive care units across the United States. It contains more than 200,000 admissions (15), with information from drug admission records, standardized care-taker notes, ventilation data, and aperiodic vital parameters, such as pulmonary artery occlusion pressure (18).

The data captured by a patient monitor is summarized in Table 1, and we describe its details in the following. A patient monitor usually displays an ECG, which records the electrical activity in the heart as electrical potential on the orders of a few millivolts. The ECG is measured using electrodes placed on the skin, whose number and position may vary. Depending on the electrode’s configuration, it is possible to have different leads, which can sometimes be monitored simultaneously. This allows detailed monitoring of the entire cardiac cycle. Additionally, heart frequency (HF), measured in bpm, reflects the fluctuations in the electrical activity of the heart using an ECG. HF should not be confused with heart rate (HR), which measures the actual contractions of the heart. HF is mostly identical to the pulse, measured in bpm, and can be detected at any peripheral body point using a pulse oximeter. This sensor also measures the peripheral oxygen saturation (SPO${}_{2}$) as a percentage. Breathing frequency (BF), expressed in $\frac{1}{\min}$, represents the respiratory rate and can also be estimated by an ECG. Furthermore, both non-invasive blood pressure (NIBP) and invasive blood pressure (IBP) can be displayed. In medical settings, both pressures are expressed in mmHg as the unit rather than the SI unit, Pa. Both include systolic pressure, i.e., the peak pressure during a heartbeat, and diastolic pressure, i.e., the minimal pressure between two heartbeats. IBP can be measured continuously, whereas NIBP is recorded only a few times per hour.

**Table 1:** Patient monitor data types.

Abbreviation	Unit	Name/description
ECG	mV	Electrocardiogram
HF	bpm	Heart frequency
Pulse	bpm	Peripheral pulse
SPO${}_{2}$	%	Peripheral oxygen saturation
BF	$\frac{1}{\min}$	Breathing frequency
NIBP	Pa or mmHg	Non-invasive blood pressure
IBP	Pa or mmHg	Invasive blood pressure

As many of the examples in this paper are based on ECG data, the fundamental components of a physiological ECG are described in the following paragraph, as introduced in Kardiovaskuläre Medizin Online (19) and Cardiovascular Medicine (20). An ECG waveform can be split into multiple segments, which are usually labeled P, Q, R, S, T, and sometimes U. These segments are shown in Figure 5. First, a small “hill” appears, called the P-wave, lasting roughly 0.1 s, followed by a horizontal line referred to as the PQ segment. The PQ segment serves as the reference or baseline for the ECG. The interval from the beginning of the P-wave to the end of the PQ segment is called the PQ interval. Afterward, a small, sharp dip labeled Q is observed, followed by a large, sharp spike, called the R-wave. This is followed by the S-wave, a downward deflection that is usually slightly deeper than Q. The two downward deflections Q and S below the baseline together with the large spike R dominate the appearance of an ECG and are often referenced together as the QRS complex. After S, the signal rises relatively smoothly to the baseline and is followed by a horizontal line called the ST segment. The beginning of this line, i.e., the point at which the ECG returns to the baseline, is called the J (junction) point. The ST segment is then followed by a T-wave. After returning to the baseline, a small U-wave might appear. The U-wave does not appear for all individuals, and its cause is not well known. The QT interval is measured from the start of the QRS complex to the end of the T-wave. It represents the time from ventricular depolarization to full repolarization. As it depends on the current heart frequency, it is usually corrected for heart frequency, which is called the QTc interval. The segment from the end of the T-wave to the start of the next Q-wave is called the TP interval. These segments and waves can be mapped onto the myocardial action potential and thus provides insights into the various stages of the cardiac cycle.

### 2.4 Anonymity

Anonymity can be seen as the absence of information that would otherwise allow the linkage of a record to an individual. One of the main tasks of anonymization is to ensure privacy by breaking such linkability while preserving the relationship between the time and value domains. Additionally, correlations between multiple domains, e.g., between blood pressure and certain cardiovascular diseases, should also be preserved. Data collected from a patient monitor exhibits strong inter-domain connections and possibly correlations. For instance, an obstruction in a tube during mechanical ventilation may be noticeable in both peripheral oxygen saturation (SPO${}_{2}$) and inspiratory pressure (P${}_{insp}$).

Even after the removal of direct identifiers, linking may of course be possible using information about an individual from the remaining data in a database record. Oprea et al. (21) demonstrated that classifications on time-continuous medical data are possible with a reasonable degree of accuracy, with a particular focus on explainable AI (XAI). In their study, the type of breath (spontaneous, mechanical, triggered) was classified using the airway flow and pressure, achieving accuracies of 79.78% for spontaneous, 81.69% for mechanical, and 77.05% for triggered breath. This automatic detection and classification can be used to extract information about an individual that is not directly provided, e.g., whether a patient was dependent on mechanical ventilation. Such a classification could be used to extract discrete information for an attack on the privacy of the individual.

To address such risks, anonymity must first be formalized, beginning with a clear definition of the goals. A key goal is to limit the risk of disclosure, as defined by Samarati and Sweeney (2) and Sweeney (22), i.e., the unintended release of explicit or inferable information about a person.

Disclosure risk can be categorized into three levels: identity disclosure, attribute disclosure, and $\rho_{1},\rho_{2}$ breaches. Identity disclosure occurs when explicit identifiers, e.g., a patient’s name or address, are present in the database or can be inferred. This inferability of identifiers might happen through external data sources, which can be linked to the record.

Another form of disclosure is attribute disclosure, which occurs when the precise value of an attribute, e.g., a patient’s diagnosis, is disclosed to an attacker. The attacker does not need to identify the victim’s record within the database to achieve this. For example, if all individuals of an equivalence class of k-anonymity share the same value for one attribute, an attacker only needs to know that the victim must be within that equivalence class to achieve attribute disclosure. Similarly, the value can be reconstructed for deterministically created overlapping regions of equivalence classes, as mentioned in Cao et al. (5).

Partial attribute disclosure can occur when the distribution of attributes in an equivalence class does not follow the distribution in the population closely. This means that an attacker learns a certain value is far more (or less) likely than others for the attribute of the victim. This was a key consideration behind the development of l-diversity by Machanavajjhala et al. (4) and t-closeness by Li et al. (3). In a $\rho_{1},\rho_{2}$ breach, neither the identity nor an attribute is disclosed to an adversary, but the belief about the victim changes fundamentally after inspecting the data, as shown by Evfimievski et al. (23).

The ambition of absolute disclosure prevention, i.e., achieving anonymity through semantic security as foreshadowed by Dalenius (24) and formalized by Goldwasser and Micali (25), is not very useful, as this anonymity cannot be achieved when additional data sources can be used by the attacker, as shown by Dwork (9). Semantic security in the context of encryption, as defined by Goldwasser and Micali (25), means that the encryption scheme is secure if no information about the plaintext can be learned by looking at the ciphertext. Similarly, Dwork (9) proposed the concept of semantic security in a privacy setting: nothing about an individual should be learnable by looking at the results of queries on an anonymized database. Thus, the corresponding privacy notion of ϵ-DP, as defined by Dwork (9), is used in this paper. The intuition is that at most ϵ is learnable about an individual when looking at the protected responses to queries on the database containing sensitive information.

### 2.5 Measuring utility

Utility quantifies the quality with which the target operation can be performed on the protected data after anonymization. More formally, it measures how good cross-attribute correlations work on an anonymized database [cf. (26)]. The necessity for an empirical metric for the utility was highlighted by Iyengar (27), LeFevre et al. (28), and Wang et al. (29). Achieving “good” utility is hard, in general, as the work to be performed on the data is often not known beforehand. If the work is known in advance, the institution responsible for anonymization can simply execute this work on the unanonymized data and release the results instead. Thus, the goal is to develop a workload-independent metric, which works for a wide range of applications. This requirement for the utility was already highlighted by Brickell and Shmatikov (26).

Utility can be measured by syntactic and semantic metrics. An example of a syntactic metric is the information loss, measured by the amount of generalization or suppression applied, as presented by Hammer et al. (30). Other syntactic metrics are presented by Machanavajjhala et al. (4), which include the mean size of quasi-identifier equivalence classes, the sum of squares of the class sizes, and the number of generalization steps performed.

Semantic metrics can be categorized as either workload-independent or workload-dependent. A workload-independent metric quantifies the “harm” done to the data by the anonymization process itself, but it does not measure the remaining utility of the data. As already mentioned, the precise workload is not known to the anonymizing party. Thus, it cannot optimize against the application-specific utility. Nevertheless, LeFevre et al. (28) proposed a workload-specific anonymization approach that aims to optimize utility for one or multiple classes of workloads.

This leads to a general definition of utility, as formalized in Definition 2.7. The methods to quantify the probabilities depend on the specific workload class; in many cases, these probabilities cannot be determined beforehand or need manual evaluation by a professional. Thus, approximations of these definitions will be needed in many instances.

DEFINITION 2.7 **(Utility)**

Given the data record e∈S of database S and the anonymization function $\alpha \;:\;D\mapsto D_{\alpha }$, which maps a data record to the anonymized data record. Due to the lack of an objectively correct decision, a decision is treated as correct if it is made based on the raw data. Respectively, any deviation between a decision made on the anonymized data and the decision made on the raw data is treated as an error. Making a decision d based on e is denoted d(e). Let f be the decision-correctness function, which returns 1 if a decision based on the raw data is the same as the decision based on the anonymized data. Thus,

$$f(d,e,\alpha )=\left\{ \begin{array}{cc} 1\; & \text{ if }d(e)=d(\alpha (e))\;\\ 0\; & \text{ else }\; \end{array}\right.$$

This leads to the definition of the utility U for a set of decisions D

$$U(D,e,\alpha )=\frac{\sum_{d\in D}f(d,e,\alpha )}{\left|D\right|}$$

Syntactic methods for privacy, such as k-anonymity (2), l-diversity [cf. (4)], or t-closeness (3), focus on obscuring the individual’s identity in the dataset by making it indistinguishable from several others. In contrast, semantic methods aim to protect the sensitive attributes themselves. For example, in the case of differential privacy (9), this is done by adding random noise to obscure any information that is specific to an individual in the dataset.

Similar to privacy, utility can also be defined syntactically or semantically. For example, the information loss is syntactical, whereas a metric that measures the accuracy of a query is semantic. In time-continuous medical applications, the syntax-based utility metric from Hammer et al. (30) measures information loss as the average width of anonymized data. This is the range of each equivalence class; it is zero for raw data and grows with anonymization unless the value fits perfectly. The raw data, e.g., an ECG, is recorded and treated as the ground truth. The information loss then quantifies the new thickness of the ECG in relation to the overall domain width. If the data is anonymized using k-anonymity for time-continuous data, as presented in Hammer et al. (30), the information loss is the average width of the equivalence class divided by the domain width.

There are usually two types of privacy models for DP: interactive and non-interactive. An interactive privacy model allows the user to submit queries to the system, which are then answered in a privacy-preserving manner. As the query can be performed on both the raw and sanitized data, it enables calculating the accuracy of each answer. The user is then given a measure of utility along with the answer to the query based on the sanitized data. In the non-interactive privacy model, the system sanitizes and releases the data to the public, thus allowing the uncontrolled execution of queries by the user post-protection.

As the releasing party does not anticipate the queries to be performed and users do not have access to the raw data, the accuracy cannot be calculated directly. Thus, only an exemplary measure of the accuracy of some previously known queries could be given to users who need access to the data. Alternatively, a utility metric can measure the preservation of patterns and trends in the data, which are needed to perform meaningful analyses and derive insight. This is highly application-specific, as the relevance of patterns depends not only on the analyzed data but also on the questions a user wants to address through the analysis.

One could think about alternatives to the proposed Definition 2.7 and come up with syntactic utility metrics. However, these approaches do not consider the underlying structure and meaning of the data. While syntactic metrics are relatively easy to implement and use, they offer limited insight into the usefulness of the anonymized data, which is the goal of a utility metric. Thus, such metrics are not well-suitable for the intended purpose. Definition 2.7 can only be applied to known and formalized queries. Furthermore, the same set of queries needs to be performed on the raw data; thus, such a definition is only feasible for interactive models or a selected set of benchmark queries performed before data release.

Non-interactive models have somewhat different requirements. As the workload is not known beforehand and Definition 2.7 can only be applied a posteriori, an alternative approach is needed for some applications. A precise calculation of the usefulness of the data to all possible classes of workload is not possible. As a result, only an estimate of the usefulness of the data is possible, which is introduced in Definition 2.8. Such a metric can also be relevant for interactive approaches, as it provides an estimation of the utility before executing queries, thus reducing the possibility of spending time and resources on the work with an unsuitable database.

DEFINITION 2.8 **(Heuristic utility)**

This definition aims to approximate utility according to Definition 2.7. Given a database S⊂D with functions $U_{p},U_{h}\;:\;S\mapsto R$, where $U_{p}$ is the utility according to Definition 2.7 and $U_{h}$ is the utility using a heuristic; then, $U_{p}(x)\approx U_{h}(x)|\forall x\subset D$.

The precise relation is often application-specific, as is the heuristic utility itself. $U_{h}$ is determined by the underlying heuristic h; however, in general, the result must be normalized similar to Definition 2.7; thus, $U_{h}(x)\in [0,1]$.

Definition 2.8 can use semantic or syntactic heuristics. Promising approaches for the approximation of utility include metrics such as accuracy, precision, and recall of a query, mean relative error (MRE), and the preservation of patterns.

The MRE is commonly defined for recorded values y and the predicted or, in our case, sanitized values $\hat{y}$:

$$\text{MRE}(y,\hat{y})=\frac{1}{N}\sum_{i=0}^{N-1}\frac{\left|y_{i}-{\hat{y}}_{i}\right|}{\left|y_{i}\right|}$$

For non-continuous data, accuracy and other relevance metrics, like precision and recall, are relatively straightforward to calculate. In the following, all necessary metrics required to adapt the MRE for use with continuous data are introduced. This adaptation could then be used to define accuracy, precision, recall, or other candidate metrics for utility.

One challenge is the quantification of distances between continuous data values, e.g., between curves. A possible candidate for such a metric is the Hausdorff distance $d_{h}(X,Y)$. Intuitively, it measures how much two subsets of a metric space X and Y must be thickened to fully contain the other (31). As an example, consider the domain of ECGs, defined over time × voltage. Let there be two ECGs, $E_{1}$ and $E_{2}$, then $d_{h}(E_{1},E_{2})$ is the minimal ϵ such that making $E_{1}$ wider by ϵ contains $E_{2}$, and vice versa. This can lead to very large values in the case of misaligned or otherwise transformed subsets, even when the general shapes are similar.

The Gromov–Hausdorff distance expands upon the concept of the Hausdorff distance by iterating over all possible isometric embeddings of two subsets into a common third space, which allows for transformations of the subsets (31). In the context of the ECG example from the Hausdorff distance, both $E_{1}$ and $E_{2}$ would be transformed into a common isometric embedding. $E_{1}$ and $E_{2}$ could be rotated or shifted. Afterward, the Hausdorff distance is calculated on these embedded $E_{1}$ and $E_{2}$. The Gromov–Hausdorff distance is defined as the infimum of these calculated Hausdorff distances over all possible embeddings.

So far, we only measure the distance between two subsets, without considering the general shape and other properties. This can be done when using the Fréchet distance, which calculates the similarity of two curves (32). Intuitively, one can imagine a dog and its owner, connected via a leash. The dog walks along curve $C_{1}$ and its owner along $C_{2}$; both can independently walk forward or stop, but neither can go back. The shortest possible leash length that allows both to reach the end of their curve is the Fréchet distance (33). This quantifies the similarity of curves.

Both the Hausdorff and Fréchet distances measure similarity, or better the lack thereof, and thus their measurements carry semantic meaning. However, they approach this from different perspectives: while the Hausdorff distance measures the proximity between sets in a metric space, the Fréchet distance measures the similarity of shape and trend between curves or trajectories. Thus, the latter is far more suitable for the intended application. However, calculating the Fréchet distance is computationally expensive. Alt and Godau (32) mentioned the runtime for an exact calculation with two polygons with p and q segments as O(pqlog⁡pq). Even the approximate version presented by Eiter and Mannila (33) has a runtime of 0(pq).

The Fréchet distance is still prone to producing high measurements due to misalignments or other transformation-induced errors. One could investigate the possibility of extending it similarly to the Gromov–Hausdorff distance, i.e., by taking the minimum overall isometric embeddings for all Fréchet distances. Still, a small number of spikes or valleys, which do not match from one curve to the other, can drastically inflate the result. This can be addressed by detecting such erroneous regions and omitting them up to a certain threshold. Then, the Fréchet distance can be calculated independently on each of the resulting segments, with the results combined to form a final similarity score. The detection of such regions and the calculation of the corresponding threshold for omission could be performed using an approximation for the geometric edit distance, similar to the one proposed by Andoni and Onak (34), in a sliding window manner or through shape matching based on the skeleton of the shape using the edit distance, as introduced by Klein et al. (35). While the approximation by Andoni and Onak (34) operates in near-linear time, it is only intended for strings. An approximation of the geometric edit distance is still somewhat expensive with O(nlog⁡n) (36). The shape matching variant by Klein et al. (35) is even more expensive. Thus, both the Fréchet distance and the Edit distance are computationally expensive.

For comparisons among multiple pairs of curves, both the basic Fréchet distance and its proposed extensions require normalization. An intuitive way to normalize for a given set of curve pairs is to calculate the sum of all distances and divide each distance by this sum.

Suitable candidate metrics to quantify the distance between trajectories along time are identified by Su et al. (37). This paper evaluates and categorizes multiple metrics for trajectories. It identifies three of them as spatio-temporal and continuous metrics. These metrics are suitable candidates for the challenges presented in this paper. Besides the Fréchet distance, the spatio-temporal Euclidean distance measure (STED), as proposed by Nanni and Pedreschi (38), was evaluated by Su et al. (37). STED quantifies the Euclidean distance between the curves along time, normalized by their length. However, this metric does not consider the shape of the curves or potential alignment or transformation issues.

In the following, we formalize one approach rigorously as an example. For this, it is assumed that the answer to a query for interactive anonymization, e.g., DP, is made based on the recorded data, and the sanitization is applied afterward. This means that both a query without anonymization and one with it applied contain the same set of curves; however, in the case of DP, the later set has added some carefully tuned noise to each curve.

In situations where this assumption is not given, the following calculation would only hold on the intersection of the sanitized and unsanitized queries. One could then provide accuracy as a tuple of this result and the number of curves that are missing in one of the sets.

To define the MRE for interactive approaches on time-continuous data, first, the difference between the curves is formalized according to Definition 2.9.

DEFINITION 2.9 **(Curve difference)**

Given a query q, the database S, the anonymization function α, and the Fréchet distance F(x,y). Additionally, let G(x,y) be the transformation analog to the one proposed for the Gromov–Hausdorff distance, which transforms x as close to y as possible. Let E(x,y,τ,ω) be the mapping, which was informally introduced in the previous paragraph. It corrects^2^ smaller regions of a mismatch from both curves x and y, with a maximum size of ω, up to a given threshold τ, using the edit distance. This leads to the definition of the difference between two curves:

$$d(c_{1},c_{2},\tau ,\omega )=F(G(E(c_{1},c_{2},\tau ,\omega ),c_{2}),c_{2})$$

A norm |∘| on curves has to be formalized to be able to adapt the MRE to time-continuous medical data. This is done in Definition 2.10. The fast Fourier transformation (FFT) maps curves to frequency space, decomposing complex waves into a structured vector of frequencies. Applying a p-norm to this vector provides a stable, mathematically consistent curve norm. Other norms could also be used; the given definition is just one candidate.

DEFINITION 2.10 **(Curve norm)**

First, the FFT is used to map the curve to a vector of its frequencies. Afterward, the p-norm for some 2≤p<∞ is applied. If FFT(c) is the function that maps a given curve to the vector of its frequencies, then the curve norm is

$$|c|=|FFT(c){|}_{p}$$

This allows defining the MRE for time-continuous data according to Definition 2.11.

DEFINITION 2.11 **(Time-continuous MRE)**

Instead of $c_{j}=\alpha (c_{i})$, $c_{i}{=}_{\alpha }c_{j}$ is used as a notation. The results of a query are noted as $R_{1}=q(S)$ and $R_{2}=\alpha (q(S))$. A normalization term for the summation is needed:

$$N_{R_{1},R_{2}}=\sum_{c_{1}\in R_{1},c_{2}\in R_{2}\;:\;c_{1}{=}_{\alpha }c_{2}}d(c_{1},c_{2},\tau ,\omega )$$

This is used to define the normalized time-continuous MRE:

$$\begin{array}{rl} & MRE(R_{1},R_{2},\tau ,\omega )=\frac{1}{N_{R_{1},R_{2}}}\\ & \;\cdot \sum_{c_{1}\in R_{1},c_{2}\in R_{2}\;:\;c_{1}{=}_{\alpha }c_{2}}\frac{d(c_{1},c_{2},\tau ,\omega )}{\left|c_{1}\right|} \end{array}$$

The components used to define the MRE on curves can also be used for accuracy, precision, and recall.

A potential approach to reduce computational complexity is to map the data into a lower-dimensional space; this is similar to SABRE-AK how uses the Hilbert space-filling curve to approximate nearest neighbors in a multidimensional feature space (7).

All these candidate metrics do not consider the semantic importance of missing or artificially introduced features. For example, for some metrics, many small changes might lead to a similar utility score, as if only a single large change, which impacts the deduced knowledge, would occur. Thus, a semantic metric should be considered, preferably one that considers application-specific requirements.

### 2.6 Measuring privacy

Besides utility, privacy is another important property of an anonymization mechanism. It quantifies the level of security an individual in the database achieves. Definition 2.12 considers not only reidentification but also the general risk an individual faces by being part of a database. According to Dwork (9), privacy is quantifies by what can be learned about an individual through data analysis, or more precisely the lack thereof. This privacy loss, e.g., with DP, is usually represented as a function of the privacy budget ϵ and the number of allowed queries to the data a.

DEFINITION 2.12 **(Privacy (9))**

For some ϵ and a, the probability of making a decision based on any attribute of the database S and an individual i is quantified by $Pr_{\epsilon ,a}(x,i)\in [0,1]$. Let the database S∖i be the same as S, but i is not included. The privacy level is

$$P_{\epsilon ,a}(i,S)=\frac{Pr_{\epsilon ,a}(S\setminus i,i)}{Pr_{\epsilon ,a}(S,i)}$$

When looking at Definition 2.12, one can note that removing the individual of interest cannot add information about them to the dataset. Therefore, $Pr_{\epsilon ,a}(S,i)\geq Pr_{\epsilon ,a}(S\setminus i,i)$; thus, the privacy $P_{\epsilon ,a}(i,S)\leq 1$. The closer the P is to 1, the less information can be gained about an individual by analyzing the data. Quantifying such probabilities remains an application-specific open task.

Now, all basic concepts and necessary notations for time-continuous anonymization have been introduced. Thus, these can be used in the following section to deduce relevant properties and analyze them formally for multiple classes of mechanisms.

## 3 Applying syntactic mechanisms to medical data

With the definitions from Section 2 and the intended application proposed in Section 1, we can now derive requirements for any anonymization mechanism intended for time-continuous medical data. Section 3.1 presents some general threats to privacy and a more detailed look into potential attacks specific to time-continuous data. The requirements are formalized to properties of the mechanisms and investigated in Section 3.2. These are then used to define some properties of anonymization mechanisms, which are investigated and proven for multiple classes of mechanisms in Section 3.3. This provides a strong formal set of properties such a mechanism must fulfill.

### 3.1 Threats to anonymity

This section discusses some general types of attacks on privacy and some specific to time-continuous data. There are multiple types of privacy threats. Reidentification is a general attack vector for anonymized data, achievable through linking or background knowledge attacks. This applies not only to time-continuous medical scenarios.

**Figure 4:**
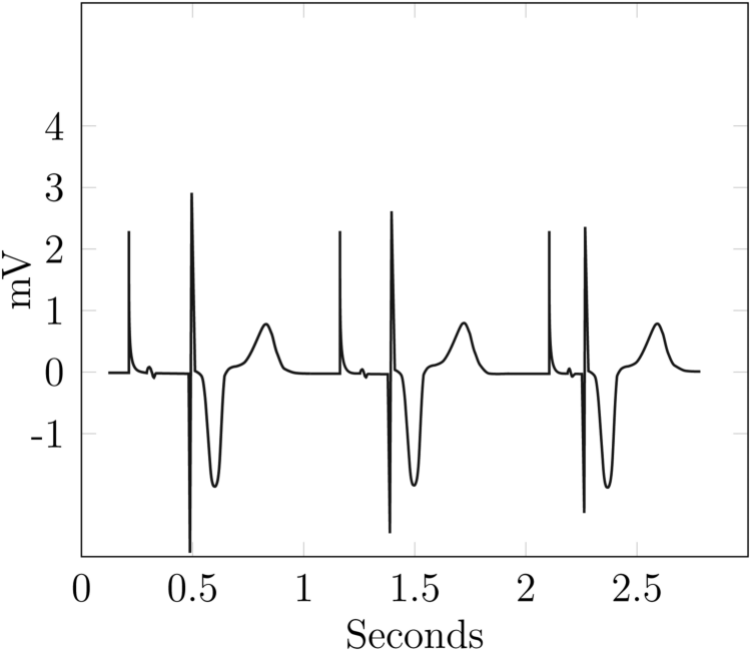
Example—ECG reidentification.

Figure 4 shows multiple cycles of an ECG from a patient with a pacemaker. The data is taken from Wikimedia Commons (39) and adapted to include a spike before the Q-wave.

**Figure 5:**
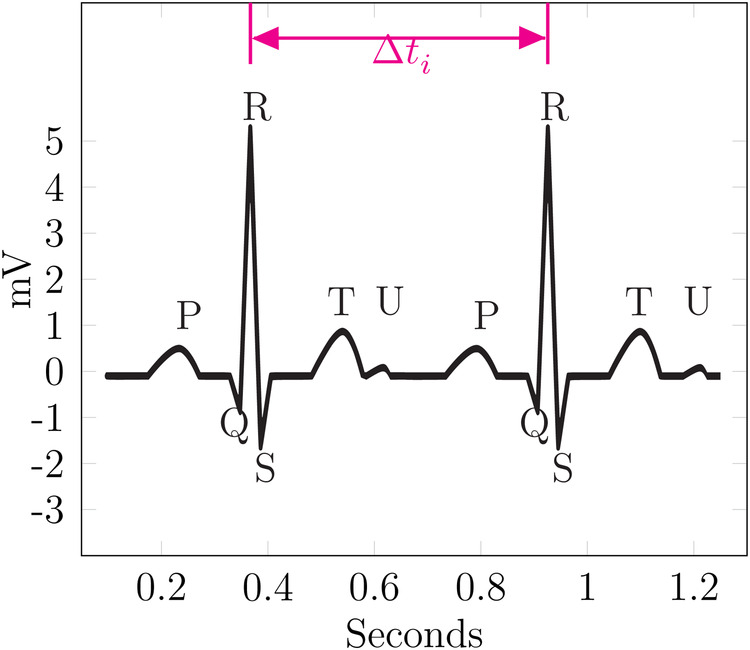
Example—Δt linking.

The ECG in Figure 4 looks rather similar to the optimal one, i.e., a typical physiological ECG in Figure 5, with most of the differences attributable to physiological and interpersonal variations. However, the additional spikes before the Q-waves are identifiable as a pacemaker with ventricular stimulation. Even without this spike, an attacker with background knowledge about their victim could identify the ECG or, at least, narrow down the set of potential targets.

Moss (40) explained how the biological sex of a patient can be determined using the ECG, which reflects the morphology of the heart. Thus, the R-wave can be used to classify ECGs by sex, as done by Nolin-Lapalme et al. (41). This can be done without any background knowledge about the target. If background knowledge is present, even more attack vectors become viable. For example, an attacker knows that their victim has a pacemaker implanted and suffers from a decreased cardiac output and feels constantly exhausted; this leads to an S–T ratio close to one or larger.

Additionally, an inference attack can be performed to statistically deduct behavioral information about an individual; again, this works for most types of data. Such an attack can be used to infer the value of an attribute or the membership of the victim to the database (42).

In the context of time-continuous medical data, reidentification could be performed by correlating different detected events or through longitudinal analysis linking multiple treatments to datasets. This enables temporal or treatment event linkage. As an example, database S with multiple records $d_{i}$ is given. Some records might belong to the same person using the aforementioned approaches, and an attacker might be able to link those records and potentially reidentify the individual.

In the following, a record i belonging to person $P_{j}$ will now be labeled as $d_{i_{P_{j}}}$. The first time-continuous data-specific approach allows correlating different detected events.

The attacker has to detect events in the record. An event is not necessarily a complex medical diagnosis but potentially something like a recurring spike in the data or the QRS part of an ECG.

An assumption is that the time spans between similar recurring events can be used to identify an individual across multiple data records. In the simplest case, $\Delta_{1}\approx \Delta_{2}$ for two detected events in $d_{1_{P_{j}}}$ and $d_{2_{P_{j}}}$. Of course, more complex patterns of Δ values can occur, which might not fully match. Even with some uncertainty, a linkage might still be possible. Thus, this temporal linkage via correlation of Δt for events presents a viable attack vector.

Figure 5 shows an exemplary ECG curve. It is based on the data from Commons (43), but a U-wave was added under the assumption that if a U-wave occurs it is usually 25% of the T-wave in amplitude (19).

In this example, $\Delta t_{i}$ is the difference between the two R-waves of the ECG. If the attacker can find the same Δt in another record, they can link those records together. Additionally, such a linkage can be verified or narrowed down, especially if multiple candidates for the linkage exist, using specific features. The existence of the U-wave, which does not occur for every person, would be good verification for the linkage.

In other words, for a database of ECGs S containing ECG e∈S and given a reference ECG $e_{a}$ known to belong to the victim, an attacker narrows down the set of potential ECGs linked to the victim. This is done by creating the set $E=\{e_{i}\;|\;\Delta t_{i}=\Delta t_{a}+\epsilon_{\Delta }\}$, where Δt is the previously defined difference between events and $\epsilon_{\Delta }$ is a tolerance, which allows some error to account for sampling variances, rounding, or potential perturbation. From these candidate ECGs, E is the subset containing the verification feature; here, the U-wave is assumed to belong to the victim. This subset is $E_{v}=\{e_{i}\;|\;e_{i}\text{ has U-wave}\}$. Depending on the chosen events and verification features, this could pose a potent attack vector.

The second time-continuous data-specific approach works similarly to the first one but detects more complex events to construct a characteristic treatment or illness pattern for a patient. This will most likely work best for more severe illnesses, with more individualized treatments, but could be combined with the first approach to work on more standard treatments.

Here, the attacker detects and classifies the events $\tau_{1_{1}},\ldots ,\tau_{1_{i}}$ for record 1 and $\tau_{2_{1}},\ldots ,\tau_{2_{j}}$ for record 2. In the best case, these sequences would match; more realistically, a metric like the edit distance must be used to determine their similarity. This allows an attacker to link multiple records together via event-based linkage and potentially track or even reidentify an individual.

**Figure 6:**
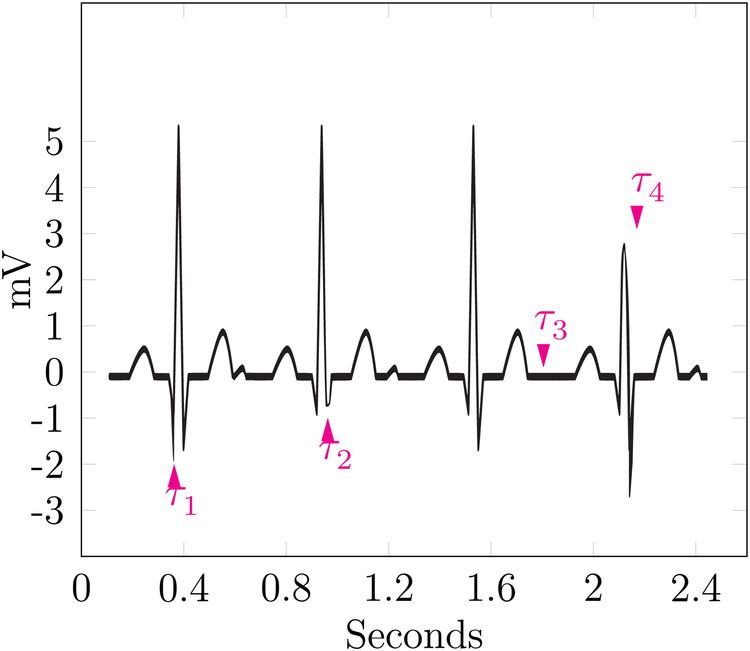
Example—event sequence linking.

Figure 6 shows four cycles of an ECG. The data is based on Commons (43). It shows four markers named $\tau_{1}$–$\tau_{4}$, each highlighting a single event.

$\tau_{1}$ marks a particularly low Q-wave, which could be an indicator for a myocardial infarction, also named Q-wave infarction (44). The second marker $\tau_{2}$ highlights a particularly shallow S-wave, as the ST complex is still facing upward; this can be seen as a non-pathological finding but is a notable event nevertheless. The highlighted event of $\tau_{3}$ is the lack of the U-wave; all cycles before and after show a significant U-wave, which is notable in itself, but its lack in just one cycle provides a strong event for identification. During the last cycle, $\tau_{4}$ points at a shallow R-wave, thus hinting at a previous myocardial infarction (13).

The sequence of these events could be used to form a signature for the patient, which is named $\sigma =\tau_{i}\tau_{i+1}\ldots \tau_{n}$. Linkage of multiple ECGs to a single known patient could then be performed by calculating the edit distance between the signatures of the ECGs and assuming them to belong to the same patient up to a certain threshold.

The feasibility of this attack vector still has to be shown and a reasonable threshold has to be found. To do this, a well-known set of events must be recognizable, which ideally should be automated.

Additionally, time patterns can be recognizable for a specific individual if only local noise is applied on the time axis. This means that the patterns are only slightly perturbed; thus, the rough date and distances between events stay intact. This could serve both as an identifier and a sensitive attribute, depending on the event and pattern.

Another possible way to infer knowledge specific to an individual from the data would be to detect certain events across multiple types of data, e.g., ECG and HF. These events are then aligned, as the detected cause for the event will happen roughly simultaneously for all types. This can lead to a restriction of the plausible parameters along the time axis and thus result in a breach of privacy for the time axis or at least increase the chances of other linking attacks.

Furthermore, biometric integration using the medical data, i.e., the use of recorded curves as a biometric identifier, could be used. The mapping of an ECG to the cardiac cycle of a person is a suitable example, as it can be used to infer characteristics about one’s cardiac parameters.

The possibility of this mapping has been demonstrated and proven successful for the authentication of users in a lab environment (45, 46). Morphological features of an identifiable in the ECG of an individual, as proposed for sex-specific differences by Moss (40), have been successfully implemented in a classifier by Attia et al. (47).

Similarly, detection mechanisms can be used to reduce the time-continuous data to discrete data. The detection of breathing cycles and breath classification provide a good example. This allows for the inference of breathing frequency and a diagnosis, which could be used in a linking attack. The feasibility of automatic detection and classification of breath types has been demonstrated by Oprea et al. (21).

Depending on the chosen noise and anonymized data, some noise might be removable, or at least the noise level could be reduced; this is particularly true if biological boundaries are not considered when applying the noise.

A wide range of potential attack vectors exist for time-continuous medical data. Some have already proven feasible, while others are introduced in this paper, requiring further investigation into their implementation and feasibility.

### 3.2 Requirements of time-continuous medical data

Time-continuous medical data comes with specific requirements w.r.t. anonymization. In the following, the requirements, which can be derived from the sanitized data alone, i.e., without inspecting the inner workings of the mechanism, are presented. To remain usable for diagnostic and research applications, the data should remain continuous and differentiable across all axes. High jumps within the data must not be introduced by any anonymization, as this will be implausible for any attacker with application-specific knowledge and thus potentially render the anonymization less powerful. The precise height of an implausible jump in the data is highly application- and data-specific and open for discussion. The sampling characteristics should be preserved. That is, any trajectory with a variable sampling rate should not be sampled with equidistant step size after anonymization. The opposite direction will most likely not be achieved, but the mean sampling rate should be preserved. For any curve, the order of the data points and the continuity of the data must be preserved. All mechanisms have to provide strong, formal privacy guarantees with a quantifiable level of protection and utility.

There are several classes of anonymization mechanisms, which are referenced later in this section and shortly introduced in the following. Additionally, the most important requirements for time-continuous medical data are evaluated. They have been compared to one another in a non-medical environment by Murthy et al. (48). The evaluated classes include generalization, suppression, masking, swapping, distortion, and perturbation. All these classes are syntactic protection methods. For some more promising classes of mechanisms, the properties forming the requirements are evaluated formally in Section 3.3.

**Generalization** replaces the existing values with semantically consistent values. It works rather intuitively for most numeric domains, as generalization to a range domain is intuitive for most applications. For categorical domains, a domain generalization hierarchy is needed, which can become unfeasible for large domains and potentially impossible if the domain has cardinality ∞ and no inherent hierarchic structure. Such mechanisms are not well suited for the tasks of anonymizing time-continuous medical data, as they fail to preserve continuity and the sampling characteristics. Additionally, the order of the data values is not preserved for such mechanisms. This is proven in Proofs 3.1.a and 3.2.a. Furthermore, generalization-based mechanisms like k-anonymity fail to provide strong formal guarantees. To our knowledge, there does not exist a generalization-based mechanism with formal, quantifiable guarantees comparable to, e.g., DP.

**Suppression**, i.e., the deletion of certain data, can be used to completely deny the usage of sensitive data. This can conceal sensitive attributes or tuples from both an attacker and a valid user. This may be a good solution if one or few records pose a very high risk of identifying an individual, as the omission of this individual can reduce the noise needed to achieve a sufficient privacy level and thus improve the utility of the anonymized data. For example, if a very rare disease is not of interest to the research question, it might be a good idea to suppress the records with this disease, as protecting them would most likely require large amounts of noise and would increase the information loss unnecessarily. This is also true for attributes of a database. That is, the removal of less relevant attributes from all records can improve utility by making the anonymization easier on the remaining database. Suppression of records alone does not provide privacy for those records that are released, but it does not affect the continuity and order of the data values, as the data itself is not changed. If attributes are suppressed, this can improve the privacy of all individuals, without affecting other attributes. Thus, such mechanisms are not suitable as a standalone solution.

**Masking**, according to Murthy et al. (48), provides similar protection to generalization and shares the same problems w.r.t. the introduced requirements. It is similar to suppression, but only parts of the data are made unusable. That is, a fraction of each attribute is replaced by a placeholder. For example, zip codes 52070 and 52062 are replaced by 520XX. This also highlights the similarity to generalization in some cases, as the two zip codes could also be generalized to 520*, where * is a wildcard character. It is easier to apply; for instance, no domain generalization hierarchy is needed to increase data protection. Additionally, each record can be masked individually and independently. Not every part of the data carries the same amount of information; for example, some regions are of high interest to identify the biological sex, as highlighted by Moss (40). Depending on the data and record, these regions vary significantly and are also likely to be interesting to the researcher. Thus, removing them is either not possible, as they would need to be identified first, or not purposeful, as these regions are the reason for releasing the data in the first place. Similar to generalization, masking does not preserve continuity and order, and to our knowledge, no approach provides strong formal privacy guarantees, with a quantifiable level of protection.

Another approach is **swapping**, which rearranges the values in each column randomly, as described by Murthy et al. (48). This works entirely independent of the type of data and without much overhead. This is easy to implement and preserves continuity and order, as the data itself is not changed. It fails to provide strong formal guarantees. The successful usage of a swapping-based approach is unlikely because the correlations across multiple domains, e.g., between ECG and diagnosis, must be preserved to maintain the usefulness of the data.

A remaining class includes **distortion**-based mechanisms. These mechanisms change the value of an attribute to something else, either by adding some noise $V_{r}$ to the value $V_{d}=V_{u}+V_{r}$, also called perturbation, or, for an explicit identifier, through a hash function (48). The latter allows for a unique reidentification within the anonymized database while not giving away the explicit identifier.

It is the most promising class for the intended purpose of this paper. Such mechanisms preserve continuity and can preserve the order if certain bounds are guaranteed. **Perturbation** is a special form of distortion and works by adding noise to the data values to ensure privacy. If such a mechanism-based approach is used, the noise must be bounded, with the bounds given by the type of data that is anonymized. For example, the noise applied to an ECG can never be in the range of full volts and seconds, as this would make it unusable. For the detection of myocardial infarctions, differences in elevation ≥0.1 mV and 80 ms width are used for the diagnosis (49, 50). Thus, the applied noise should be substantially lower. Additionally, the added noise is not independent for each point, as highlighted by Cao et al. (51). Therefore, the context of the curve must be considered when adding noise. Perturbation does preserve the continuity of the data. The preservation of the order is not guaranteed unless the noise is sufficiently bounded. This is proven in Proofs 3.1.b and 3.2.b.

**Figure 7:**
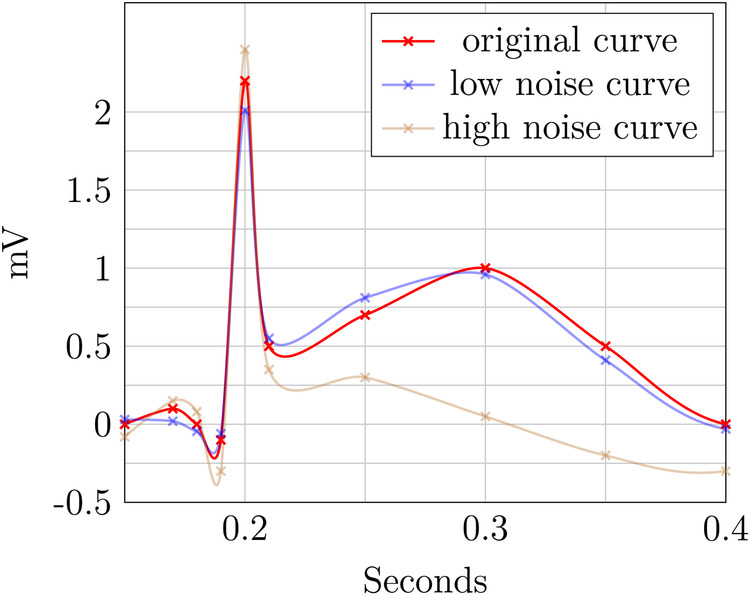
ECG perturbation—value axis.

**Figure 8:**
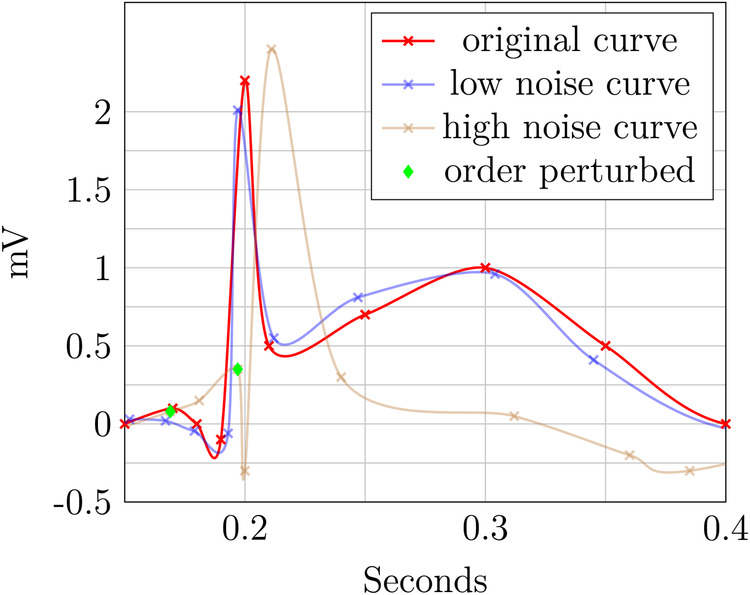
ECG perturbation—time and value axes.

The two perturbed ECG plots in Figures 7 and 8 illustrate the privacy–utility tradeoff in time-continuous data and problems arising when applying perturbation-based approaches to such data. The data segment is loosely based on ExCard Research (52). Figure 7 adds noise only to the value axis, while Figure 8 adds noise to both time and value axes. In both figures, the red curve is the original, the blue curve has a small amount of added noise, and the brown curve has a larger amount of noise added. Laplacian noise with an absolute maximum of 0.5 s, 1.9 mV perturbation and a mean perturbation of 0.289 s, 0.75 mV is added to the low-noise version. For the high-noise version, up to 1.5 s, 9.5 mV is added, and on average, each sample is perturbed by 1.05 S, 2.86 mV.

Even when perturbing both axes, the slight distorted shape of the signal in the blue curve retains its fundamental diagnostic features and temporal order. In contrast to this, the highly perturbed version—displayed in blue—is usable in Figure 8. The order is retained, but the characteristically raised T-wave is drastically damped in this example. Still, the elevated T-wave visible hints at signs of subacute myocardial infarction, even though it is an unusual shape, potentially obscuring. For the version in Figure 8, the ECG becomes completely unusable, as the order is destroyed and no meaningful insight can be drawn from the remaining data. The highly perturbed version in Figure 8 lead to a change in the order of the samples. This example highlights that an independent, pointwise application of perturbation is not useful for such data.

Multiple perturbation-based approaches exist that provide strong formal guarantees, e.g., DP. It is based on carefully adding crafted noise to the data to distort it as much as needed to fulfill the privacy guarantees. That is, the approach must stay within the budget of leaked information, thus reducing the risk to each individual to a quantifiable amount. This can be done both interactively and non-interactively. To our knowledge, there is no DP-based approach that focuses on time-continuous data, especially in a medical environment, even though the risk of privacy leakage through time-continuous data was identified and quantified by Cao et al. (51) as temporal privacy leakage. The properties of DP are formally evaluated in Proofs 3.1.c and 3.2.c.

Alternative approaches like PrivECG (41), which employs a GAN to improve the privacy of an ECG w.r.t. the classifiable of the biological sex, are promising but fall short of formally quantifying the level of privacy. The only privacy metrics evaluated against this approach are chosen accuracy metrics of a classifier with and without PrivECG in place. While this provides good initial intuition, it makes no statement about the information-theoretic level of privacy, as DP would, and only considers one specific classifier.

In summary, a mechanism addressing the anonymization of time-continuous medical data must preserve continuity and order. Where possible, the step-size characteristics should be preserved. Spatio-temporal correlation is of high importance to most analyses and must therefore be preserved. Such a mechanism must provide a strong formal foundation and a quantifiable level of privacy. None of the syntactic approaches provide such guarantees. Thus, a DP-based approach seems to be the most promising candidate, even though it introduces potentially fake data.

### 3.3 Properties of anonymization mechanisms for time-continuous medical data

This subsection defines some properties of anonymization mechanisms that are relevant for time-continuous medical data and proves whether these properties hold for certain classes of mechanisms.

If the requirements for continuity are fulfilled, a dataset is continuous according to Definition 2.5; this property should also hold after applying anonymization. This might be true for some classes of anonymization approaches, while others might fail to provide such a guarantee, as proposed in Theorem 3.1.

THEOREM 3.1 **(Continuity preservation of time-continuous anonymization)**

Some classes of anonymization algorithms are inherently safe w.r.t. the continuity, as defined in Definition 2.5 and Corollary 2.1, while others might lose the continuity through anonymization. The proof of this theorem follows from Theorems 3.1.a and 3.1.b and could be extended to other classes.

The intuition behind Theorem 3.1.a and the corresponding proof is that the generalization of a time-continuous domain is not necessarily an endomorphism. That is, the domain after the anonymization can differ from that of the raw data. This can cause a loss of continuity, as shown in the following.

THEOREM 3.1.a **(Continuity preservation of time-continuous generalization)**

Generalization-based approaches are not inherently safe w.r.t. preserving the continuity of the anonymized data.

PROOF OF THEOREM 3.1.a. Given a domain D, its generalized domain $D_{g}$, and the anonymization function $g\;:\;D\mapsto D_{g}$. If a dataset in D is continuous according to Definition 2.5, there must exist the subtraction ⊙−⊙, absolute |⊙|, and order ⊙<⊙ operator on its values, and the last has to define a total order on all non-negative elements of the domain. Additionally, there exists the interpolate f, as defined in Definition 2.5, such that, for every point in the time domain, there is a mapping to some value in D.

For $D_{g}$, these operators must also exist; otherwise, Definition 2.5 is not fulfillable. This is not necessarily the case for every generalized domain.

There indeed exists such a domain that D is continuous, and after applying g, the continuity is lost. This means that $D_{g}$ is not continuous according to Definition 2.5.

Let D be the domain of an ECG. That is, the domain of each data point is T×Q, where the unit of the second element of the tuple is mV. Both seconds and rational numbers have a straightforward subtraction, absolute, and order operation. Additionally, both can be interpolated, e.g., using Equation 2, such that the domains are indeed continuous.

Let g map each data entry, that is, every curve, to the corresponding diagnosis. Then, the value part of the generalized domain, called $I_{g}$ for **I**llnesses **g**eneralized, is a nominal. While it could be the ICD-Coding, the following simplification is used for the sake of readability:

$$\begin{array}{rl} I_{g} & =\{Myacadric\;Infarction\;(MI),\\ & \;Cardiac\;Arrhythmia\;(CA),\\ & \;Other\;Illness\;(OI),\;Healthy(H)\} \end{array}$$

Then, the generalized domain is $D_{g}=T_{g}\times I_{g}$, where $T_{g}$ is the generalization of the time domain, e.g., up to a day. In such a case, there exists no interpolate between values nor is there a meaningful subtraction or absolute operator in $I_{g}$, as these are nominal values. Thus, the ϵ,δ criterion, according to Definition 2.5, is not fulfillable, and $D_{g}$ is not continuous, even though the non-anonymized domain D has this property. Therefore, generalization-based approaches are not inherently safe w.r.t. the preservation of the continuity of the anonymized data.

Intuitively, Theorem 3.1.b means that perturbation does not change the domain of the data. This is rather straightforward, as adding some noise to data of this domain cannot change the domain but only the data value. Thus, continuity will always be preserved.

THEOREM 3.1.b **(Continuity preservation of time-continuous perturbation)**

In contrast to Theorem 3.1.a, the anonymization mapping of a perturbation-based approach is the endomorphism p:D↦D with domain D, as anonymization only applies some form of noise to the data.

PROOF OF THEOREM 3.1.b. As the domain is continuous before anonymization and does not change through the mechanism, the dataset remains continuous after applying p. If dataset d from D is continuous, the perturbed dataset $d_{p}$, which is produced by applying p to every element of the dataset, is also continuous. This is because continuity is an attribute of the domain, which has not changed. Thus, perturbation is inherently continuity preserving w.r.t. Definition 2.5.

As an example of semantic approaches, differential privacy is investigated. For the properties of preservation of continuity and order, it does not matter if the approach is interactive or non-interactive, as the mechanisms that provide privacy are the same. In interactive differential privacy, a user of the mechanism submits a query, and a custom-tailored response is given to that query, which represents the database w.r.t. the statistical properties, adheres to the privacy budget, and tries to answer the query as well as possible. Thus, some carefully crafted noise is added to some subset of the database; the amount of noise might vary depending on the attribute. That is, for a query interested in heart-related issues, adding larger amounts of noise to the age might be acceptable, while the ECG values are less perturbed. For the non-interactive variant, the amount of noise is decided beforehand and the database is released as a whole. This can lead to different distributions and levels of noise, but the general mechanism is the same and thus both variants can be investigated together. It should be noted that DP, as described in Theorem 3.1.c, means the application of DP to time-continuous data that each time entry is treated independently. This comes with certain privacy implications, as demonstrated by Cao et al. (51).

Similar to perturbation-based approaches, Theorem 3.1.c can be explained intuitively by the fact that the domain itself is not changed by anonymization. This kind of noise is not important for the preservation of continuity.

THEOREM 3.1.c **(Continuity preservation of differential privacy)**

Similar to Theorem 3.1.b, anonymization mapping of a differential privacy-based approach is endomorphism p:D↦D with domain D, as anonymization applies carefully crafted noise to the data.

PROOF OF THEOREM 3.1.c. Proof 3.1.b also applies to Theorem 3.1.c, as both classes of privacy mechanisms have an endomorphism as their anonymization, and this is the only prerequisite for this proof. Thus, DP is inherently continuity preserving w.r.t. Definition 2.5.

For many applications, especially in a medical environment, time serves as more than just a means of ordering data points. It functions as a continuous axis, which contributes to the joint meaning of the data. For example, the spacing between measurements carries additional information regarding the level of trust one can have within a given segment in the curve; thus, Definition 2.4 is needed.

A generalization along the time axis would lead not only to information loss due to reduced precision regarding the time value but also possibly to loss of information about the local sampling rate. Such an approach still preserves the ordering of the data. A perturbation-based approach will threaten not only the precision and local sampling rate but also the ordering. Thus, further restrictions are needed for the intended usage to ensure that the order is preserved. For example, a bound could be introduced to the level of noise, which is applied to the time axis. The ordering property is investigated in Theorem 3.2.

THEOREM 3.2 **(Order of time-continuous anonymization)**

Some classes of anonymizing algorithms pose a threat to the order of time-continuous data, whereas others are inherently safe in this regard. The proof follows from Theorems 3.2.a and 3.2.b and could be extended to other classes.

THEOREM 3.2.a **(Order of time-continuous generalization)**

Generalization-based approaches are inherently safe w.r.t. the order of time-continuous data.

PROOF OF THEOREM 3.2.a. Given the records $d_{i}=(t_{i},v_{i})\geq_{t}$ and $d_{i+1}=(t_{i+1},v_{i+1})$, where $\geq_{t}$ is the total order w.r.t. the time entry. Then, a generalization-based approach might group together records $d_{i},d_{i+1},\ldots ,d_{j}$ with $d_{i}\geq_{t}d_{i+1}\geq_{t}\ldots \geq_{t}d_{j}$ into the non-overlapping generalizations $g_{1}=(t_{g_{1}},v_{g_{1}})$ and $d_{k},d_{k+1},\ldots ,d_{l}$ with $d_{k}\geq_{t}d_{k+1}\geq_{t}\cdots \geq_{t}d_{l}$ to $g_{2}=(t_{g_{2}},v_{g_{2}})$. Any generalization based on the closeness of the time value would lead to $g_{1}\geq_{t}g_{2}$ if and only if $d_{j}\geq_{t}d_{k}$ and $g_{2}\geq_{t}g_{1}$ otherwise, thus preserving the ordering.

**Figure 9:**
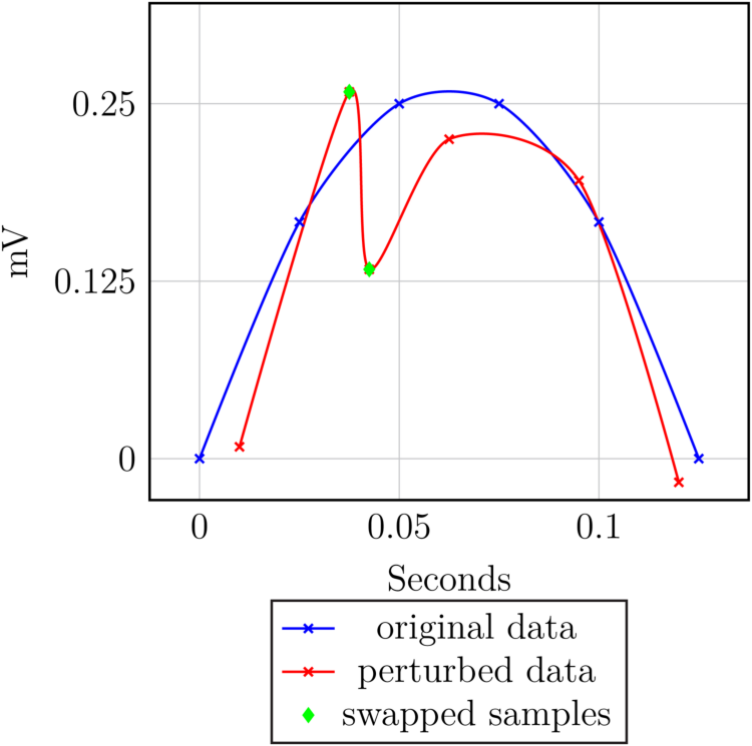
Order perturbation example.

Intuitively, if too much noise is added to the time axis, some samples might be swapped. Thus, the order is not preserved; this is visualized by an example in Figure 9 and is formalized in Theorem 3.2.b and Proof 3.2.b.

THEOREM 3.2.b **(Order of time-continuous perturbation)**

Perturbation-based approaches are not inherently safe w.r.t. the order of time-continuous data.

PROOF OF THEOREM 3.2.b. Given the records $d_{i}$ and $d_{k}$ and the corresponding perturbation values $p_{i}$ and $p_{k}$, where $p_{j}=(t_{\;p_{j}},v_{\;p_{j}})$ produced by the perturbation mechanism. Then, the perturbed data points are $d_{i}^{\prime }$ and $d_{k}^{\prime }$ with $d_{j}^{\prime }=d_{j}+p_{j}=(t_{j}+t_{\;p_{j}},v_{j}+v_{\;p_{j}})$. If $t_{i}>t_{k}$ and $t_{\;p_{k}}>(t_{i}-t_{k})+t_{\;p_{i}}$, then $t_{k}+t_{\;p_{k}}>t_{i}+t_{\;p_{i}}$. Thus, $d_{i}\geq_{t}d_{k}$ holds, but the data gets perturbed such that $d_{k}^{\prime }\geq_{t}d_{i}^{\prime }$ holds. That is, the ordering is not preserved.

As Theorem 3.2.b highlights the threat to the order of the data, any perturbation-based approach intended for time-continuous data has to employ a process of adding noise to the data designed with this safety in mind to preserve the order of time-continuous data. That is, the added noise p has to be sufficiently bounded to preserve the order.

THEOREM 3.2.c **(Order of differential privacy)**

Differential privacy-based approaches are not inherently safe w.r.t. the order of time-continuous data.

PROOF OF THEOREM 3.2.c. Similar to continuity, the order property of DP can also be proven by referencing the perturbation-based approaches. This is the case, as the proof only depends on the mechanism adding noise along both the value and time axes, with no hard guarantee for the amount of noise being added on a single point. Thus, by extension of Proof 3.2.b, the ordering is not preserved.

Similar to perturbation-based approaches in general, Theorem 3.2.c shows the threat to the data order. Adding noise along both axes can lead to a loss of order; thus, a carefully crafted mechanism for time-continuous data has to be designed with this safety in mind to preserve the order of time-continuous data for DP.

The intrinsic dependency on time-continuous data implies that any approach aiming to anonymize such data should not only preserve the ordering, as shown using Theorem 3.2, but also the continuityas defined in Definition 2.5, and possibly the characteristics of the step time, as defined in Definition 2.4.

## 4 State of the art

Samarati and Sweeney (2) proposed the distinction between explicit identifiers, quasi-identifiers, and sensitive attributes and the mitigation of certain privacy attacks using k-anonymity. It aims to reduce this risk by grouping together k data records and generalizing the quasi-identifiers (2, 22). T-closeness reduces this risk of distribution attacks by introducing a constraint that forces the distribution of sensitive attributes within a grouping to be at most t apart from the population distribution (3). This makes the usage of l-diversity, as proposed by Machanavajjhala et al. (4), obsolete, as pointed out by Li et al. (3).

CASTLE introduces an anonymization scheme based on k-anonymity for continuous streams of discrete data, see Cao et al. (5, 6). This was adapted to a t-closeness first approach with SABRE by Cao et al. (7). Both approaches fail to preserve the continuity of the data. All approaches based on k-anonymity share the same basic problem: There is no way to mathematically determine which attribute is a quasi-identifier and which is a non-identifying sensitive attribute (26). This leads to a lack of provable privacy, making them unsuitable for time-continuous medical data. Information-theoretic approaches like DP (9) achieve this formal level of anonymization and do not build upon a vague definition of quasi-identifiers.

Cao et al. (51) demonstrated that point-by-point anonymization with DP, which is common, poses the risk of information leakage through temporal correlations between values. This was formalized as the temporal privacy leakage. The attack vector was viable in the performed experiments and thus posed the risk to one’s privacy via spatio-temporal or continuous data.

Nergiz et al. (8) presented an approach for anonymization of trajectories, which preserves the spatio-temporal relation. It employs a group-and-link approach and anonymizes the data by releasing a representative constructed by choosing a random representation point in each group. While this approach preserves the spatio-temporal relation and order of the points by grouping non-overlapping areas of points, it does not preserve the data truth, as the representative might look nothing like any of the trajectories it represents because each representation point is generated independently and randomly. This can also cause large jumps in the representative, which threatens the continuity of the data.

Dankar and El Emam (10) reviewed the specific requirements for applying DP to health data, stipulating that it should be efficient, provide strong privacy guarantees, and exhibit adaptability. For the interactive approach, it should facilitate a wide range and a large number of queries. In the non-interactive case, special requirements are presented, as medical professionals and biomedical researchers like to “look at the data”; that is, explorative research is requested. As it is usually not their main task, many try to avoid changes in the processes and tools they have become used to, as they do not have the time to put much effort into changing and adapting them. A good utility for a wide range of queries is possible. This also holds for a non-interactive approach. However, besides the technical difficulties, a significant challenge lies in the social factor of a heterogeneous user group, which is reluctant to technical changes.

Olawoyin et al. (11) presented a novel approach for anonymizing spatio-temporal patient data. This approach aims to protect the temporal attributes, with a five-level temporal hierarchy and temporal representative points, while applying DP with Laplacian noise to the spatial attributes. The spatio-temporal relation is preserved by means of temporal representative points. Due to the very coarse generalization in the temporal hierarchy, this is not suitable for continuous trajectories. For instance, the order of data points becomes ambiguous.

A k-anonymity-based mechanism with t-closeness for time-continuous medical data was developed and successfully evaluated by Hammer et al. (30). This mechanism uses the Fréchet distance to calculate the similarity between curves and the information loss as a utility metric. The data is optionally split along the time axis. This reduces the computational load drastically while having a positive impact on the utility of the evaluated datasets. It falls short of providing strong formal guarantees for privacy, as it is based on k-anonymity.

A novel method to provide privacy for ECGs employing generative adversarial networks (GANs) is proposed by Nolin-Lapalme et al. (41). This method aims to prevent the reidentification of biological sex based on the publication of the ECG. To achieve this, it is shown that a 12-electrode clinical ECG is indeed suitable to distinguish sex.

According to Becker (53), the R-wave is the most salient feature to classify an ECG according to sex. This morphological distinction of the heart is further explained by Moss (40).

Additionally, the rising interest in ECG data, especially for biometric authentication by Melzi et al. (54) and data sharing by Flanagin et al. (55), was highlighted by Nolin-Lapalme et al. (41). There already exist large ECG databases such as PhysioNet/CinC (56), PTB-XL (16), and large-scale medical databases like MIMIC IV (14), which enable the development and testing of such an approach without having to measure the records beforehand.

Especially, with the rise of artificial intelligence applications in recent years, the threats to one’s privacy are also rising, especially in a medical environment (57). The viability of such an attack has already been described by Attia et al. (47), who used a CNN to determine the biological sex and age. It resulted in a 90.4% accuracy for the sex classification and an average error of 6.9±5.6 years for the age.

PrivECG, a CNN similar to that developed by Attia et al. (47), forms the basis of the evaluation of the approach of Nolin-Lapalme et al. (41). PrivECG aims to limit the classifyability of ECGs, thus increasing privacy. The original version of PrivECG resulted in a sex prediction accuracy of 0.686±0.012 vs. the original 0.882±0.022. This was further improved by PrivECG λ, resulting in a sex prediction accuracy of only 0.529±0.014 after sanitation, practically making the prediction impossible. There are some limitations to PrivECG. Nolin-Lapalme et al. (41) noted that an interaction between diseases and sex can exist, meaning a prediction of a disease could lead to an accurate prediction of the sex. Similarly, other attributes, which might be encoded in the ECG like age or ethnicity, remain unaffected by PrivECG (41). Additionally, it should be noted that no quantification of the achieved privacy level, like the case when using DP, can be given with PrivECG. The evaluation only compares the accuracy of one specific CNN on the original and sanitized databases. This might not generalize to other attack vectors and is certainly not based on any information-theoretic guarantees.

Furthermore, there are no remarks on general privacy, e.g., the risk of reidentification or linking attacks. PrivECG only aims at preventing sex classification of an ECG. Nolin-Lapalme et al. (41) described many metrics for the use in their algorithm and its evaluation. They proposed some general metrics, like the F1-score, i.e., the harmonic mean of the precision and recall, or the root mean square error. They also suggested using the Fréchet distance (32) as a measure of the difference between ECGs before and after sanitation. However, they did not evaluate this idea further.

The Fréchet distance was used as a similarity metric for different time-continuous medical datasets by Hammer et al. (30). Additionally, a few ECG-specific metrics of similarity were introduced, e.g., the average mean difference from the baseline or the average standard variation in R-wave amplitude for a single ECG cycle (41). However, further research on the viability of these metrics is needed to asses their usefulness.

Kaissis et al. (58) evaluated the application of privacy-preserving mechanisms to medical data, especially medical image data. They highlight that current approaches are often insufficient and risk patient privacy, making collaboration or sharing of data challenging. The increasing utilization of data, especially using AI in areas like medical image processing, can provide large benefits to medical professionals and patients. To achieve these benefits without sacrificing one’s privacy, suitable privacy mechanisms are needed. DP is identified as a suitable candidate.

It is noted that the specifics of DP implementation for medical image data remain unclear (58). Qayyum et al. (59) provided an overview of the need for privacy and security in medical data settings and proposed some well-known solutions. They identified multiple approaches to ensure safety, clarify prediction causality, and reduce the risk of certain attack types. Additionally, DP is identified as one of the most promising candidates to provide privacy in medical data sharing and machine learning applications (59). While this paper does not address medical data-specific issues with DP, it does reference Beaulieu-Jones et al. (60) as an example of privacy-preserving machine learning in a medical environment.

Beaulieu-Jones et al. (60) presented an application of DP with cyclical weight transfer to the eICU database (15) and the Cancer Genome Atlas (TCGA) (61). From neither database, time-continuous data is used.

Beaulieu-Jones et al. (60) showed that integrating DP into the training of a machine learning model in a medical environment is feasible. However, it was limited to discrete data, meaning that their developed process cannot be applied directly to time-continuous data.

In summary, many anonymization approaches exist, most of which are not suitable for continuous or medical data. Some might be extendable to fit the specific needs, but none seem acceptable as is.

## 5 Conclusion

The spatio-temporal structure of continuous medical data is essential for its utility, especially in diagnostic and predictive applications. However, existing anonymization approaches that aim to preserve this structure often fall short when applied to time-continuous data.

In this work, we discussed the precise requirements for anonymizing such data and evaluated different classes of mechanisms with respect to these requirements (Section 3.2). We also elaborated on a set of necessary properties for any anonymization technique in this domain (Section 3.3), highlighting the challenges of meeting strict privacy standards while maintaining clinical utility.

Medical data imposes particularly stringent demands on anonymization due to both the sensitivity of the information and the high risk to individuals in the event of a privacy breach. Unlike domains where damages can be mitigated post-breach, e.g., financial restitution in a banking scenario, medical data, once leaked, cannot be retracted. Consequently, anonymization mechanisms must rely on strong formal foundations capable of supporting provable and quantifiable privacy guarantees.

A key challenge in this context is the privacy–utility tradeoff. While a higher level of privacy provides stronger protection against misuse and inference attacks, it often comes at the cost of reduced data utility. This is particularly problematic in medical domains where diagnostic accuracy or model performance can be critically dependent on subtle temporal patterns in the data. Moreover, utility metrics tend to be domain-specific; for instance, a metric suited to evaluating ECG data for sinoatrial node disorders might be inadequate for myocardial infarction detection. Therefore, any useful anonymization scheme must strike a careful balance between minimizing information loss and ensuring robust privacy guarantees.

Importantly, insufficient privacy is not only problematic in cases of outright data breaches—it can also compromise individuals’ rights even when data is accessed by authorized parties. The trust patients place in medical professionals does not necessarily extend to insurers or third-party entities. Thus, any anonymization method must respect contextual integrity and consent-based data sharing.

To address these challenges, we advocate for a version of DP adapted specifically to time-continuous medical data. By incorporating temporal correlation handling, as highlighted in (51), and preserving order and continuity (Theorems 3.1.c, 3.2.c), such a mechanism could offer a principled approach to managing the privacy–utility tradeoff. Specifically, the noise addition process must be context-aware—i.e., informed by preceding and subsequent data points—to minimize the impact on utility while preserving privacy.

The development of such an adapted DP mechanism holds great promise. It could facilitate secure sharing of medical datasets beyond the originating institution, enable more effective training and validation of AI models, and ultimately lead to better clinical outcomes. A robust anonymization framework that respects both patient privacy and the needs of medical research has the potential to unlock significant progress—ethically, legally, and scientifically.

## Data Availability

The original contributions presented in the study are included in the article/supplementary material; further inquiries can be directed to the corresponding author/s.

## Footnotes

^1^The operators are needed for the ϵ,δ criterion.

^2^Removes, inserts, or changes the value.
